# Genetic and Biochemical Characterization of the Cell Wall Hydrolase Activity of the Major Secreted Protein of *Lactobacillus rhamnosus* GG

**DOI:** 10.1371/journal.pone.0031588

**Published:** 2012-02-16

**Authors:** Ingmar J. J. Claes, Geert Schoofs, Krzysztof Regulski, Pascal Courtin, Marie-Pierre Chapot-Chartier, Thomas Rolain, Pascal Hols, Ingemar von Ossowski, Justus Reunanen, Willem M. de Vos, Airi Palva, Jos Vanderleyden, Sigrid C. J. De Keersmaecker, Sarah Lebeer

**Affiliations:** 1 Centre of Microbial and Plant Genetics, K. U. Leuven, Leuven, Belgium; 2 INRA, UMR1319 Micalis, F-78350 Jouy-en-Josas, France; 3 AgroParisTech, UMR Micalis, F-78350 Jouy-en-Josas, France; 4 Institut des Sciences de la Vie, UCL, Louvain-la-Neuve, Belgium; 5 Department of Veterinary Biosciences, University of Helsinki, Helsinki, Finland; 6 Laboratory of Microbiology, Wageningen University, Wageningen, The Netherlands; 7 Department of Bioscience Engineering, University of Antwerp, Groenenborgerlaan 171, Antwerp, Belgium; University of Kansas Medical Center, United States of America

## Abstract

*Lactobacillus rhamnosus* GG (LGG) produces two major secreted proteins, designated here Msp1 (LGG_00324 or p75) and Msp2 (LGG_00031 or p40), which have been reported to promote the survival and growth of intestinal epithelial cells. Intriguingly, although each of these proteins shares homology with cell wall hydrolases, a physiological function that correlates with such an enzymatic activity remained to be substantiated in LGG. To investigate the bacterial function, we constructed knock-out mutants in the corresponding genes aiming to establish a genotype to phenotype relation. Microscopic examination of the *msp1* mutant showed the presence of rather long and overly extended cell chains, which suggests that normal daughter cell separation is hampered. Subsequent observation of the LGG wild-type cells by immunofluorescence microscopy revealed that the Msp1 protein accumulates at the septum of exponential-phase cells. The cell wall hydrolyzing activity of the Msp1 protein was confirmed by zymogram analysis. Subsequent analysis by RP-HPLC and mass spectrometry of the digestion products of LGG peptidoglycan (PG) by Msp1 indicated that the Msp1 protein has D-glutamyl-L-lysyl endopeptidase activity. Immunofluorescence microscopy and the failure to construct a knock-out mutant suggest an indispensable role for Msp2 in priming septum formation in LGG.

## Introduction

The ∼75-kDa (LGG_00324 or p75) and ∼40-kDa (LGG_00031 or p40) Major secreted proteins are the two most abundant proteins found in the spent culture supernatant of probiotic *Lactobacillus rhamnosus* GG (LGG). These proteins are designated here Msp1 and Msp2 respectively. Previously, these proteins were demonstrated to prevent cytokine-induced apoptosis and promote intestinal epithelial cell homeostasis [1], [2]. More recently, recombinant Msp2 (p40) was shown to prevent and treat experimental colitis by an epidermal growth factor receptor-dependent mechanism [3]. Interestingly, although the Msp1 and Msp2 proteins both show homology to cell wall hydrolases [1], the physiological function associated with such an enzymatic activity has not yet been elucidated in LGG.

The cell wall of Gram-positive bacteria is mainly composed of a thick layer of peptidoglycan (PG), an amino-sugar polymer cross-linked with peptide bridges. This multilayered murein sacculus, in addition to being responsible for maintaining cell shape and morphology, helps to protect bacteria from the external and internal osmotic stresses that can cause cell lysis [4]. The sugar component of PG consists of alternating β-(1,4)-linked *N*-acetyl-glucosamine and *N*-acetyl-muramic acid. The peptide side chain of three to five amino acids is attached to *N*-acetyl-muramic acid. The exact constitution of the peptide side chain and cross-links is species-specific. Cell wall hydrolases catalyze the cleavage of PG sugar or peptide chains. Based on their cleavage specificities, cell wall hydrolases can be divided in *N*-acetyl muramoyl-L-alanine amidases, carboxypeptidases, endopeptidases, *N*-acetylglucosaminidases and *N*-acetylmuramidases [5]. These cell wall hydrolases play important roles in the regulation of cell wall growth, turnover and maintenance, and in the separation of daughter cells [6]. Thus, besides their hydrolytic specificity, cell wall hydrolases also differ by their site of action (e.g. at septum or whole cell wall) and timing of action (e.g. during cell division or during growth for turnover and maintenance) [6].

Here, by using a combination of genetic and biochemical approaches, we investigated Msp1 and Msp2 for their role as potential cell wall hydrolases in LGG.

## Results

### Inactivation of msp1 leads to a defect in cell separation

**Figure 1:**
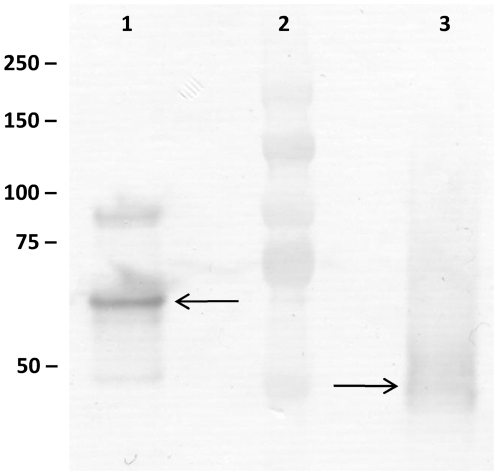
Western blot using Msp1 antiserum on purified supernatant samples of LGG wild-type culture (lane 1) and of the *msp1* mutant CMPG10200 (lane 3). Lane 2 is the Kaleidoscoop molecular weight marker (Bio-Rad).

**Figure 2:**
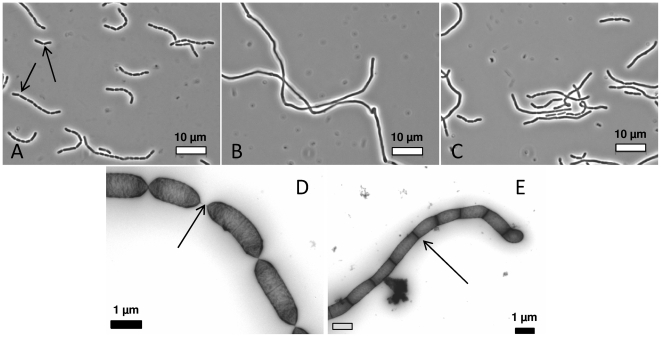
Phase-contrast (A,B&C) and Transmission Electron Microscopy (TEM) pictures (D&E) of LGG wild-type (A&D), *msp1* mutant cells CMPG10200 (B&E) and plasmid-complemented CMPG10203 cells (C). Based on the TEM data, the average length of the *msp1* mutant cell chains was estimated to be 5 to 10-fold longer than the wild-type cell chains. Arrows indicate sites of cell septa.

To investigate the biological roles of Msp1 and Msp2 in live bacteria, a knock-out mutant analysis was first chosen. An *msp1* knock-out mutant, designated CMPG10200, was constructed by insertional mutagenesis. Absence of full length Msp1 was confirmed by Western blotting using anti-Msp1 antiserum (Figure 1). As a result of the insertional mutagenesis strategy used, a truncated Msp1 protein (∼50 kDa) that lacks the C-terminal NlpC/P60 domain can be detected in the supernatant of the *msp1* mutant. This mutant is able to grow to wild-type cell densities (data not shown), but shows a clear defect in cell separation as visualized by phase contrast and electron microscopy (Figure 2). Microscopic examination of the *msp1* mutant showed the presence of rather long and overly extended cell chains (5 to 10 times longer than in LGG wild-type), suggesting that normal daughter cell separation is hampered. Plasmid-mediated complementation of the *msp1* knock-out mutant restored the wild-type phenotype (Figure 2C). Overnight cultures of the *msp1* mutant strain (in MRS or AOAC medium) also revealed major aggregation clusters, a phenomenon not observed with LGG wild-type cells. Complementation restored the wild-type phenotype (data not shown). Various attempts for the construction of an *msp2* knock-out mutant failed (see materials and methods), which suggests an indispensable role for the Msp2 protein in the viability of LGG.

### Cellular localization of Msp1 and Msp2 by immunofluorescence microscopy

**Figure 3:**
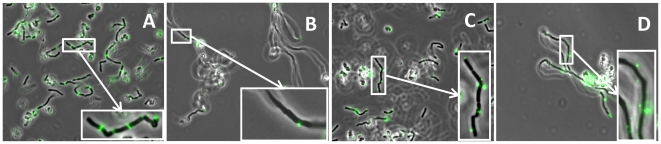
Indirect immunofluorescence microscopy (A,B,C&D). Anti-Msp1 (A&B) and anti-Msp2 (C&D) rabbit antisera were used on wild-type (A&C) en *msp1* mutant cells (B&D). Anti-rabbit IgG antibodies conjugated with Alexa Fluor 488 (Invitrogen) were used to visualize the Msp1 and Msp2 localization on the cells.

To further characterize the specific site of action, indirect immunofluorescence microscopy with anti-Msp1 or anti-Msp2 serum was used to analyze cell samples taken at various time points during the growth phase of wild-type LGG. This revealed that Msp1 is localized near the mature septa of exponential cells (Figure 3A). No fluorescence was detected in exponentially grown *msp1* mutant cells stained with anti-Msp1 serum (Figure 3B). With the same approach, the Msp2 protein was also found to be localized at the cell wall of LGG, often as two fluorescent spots near emerging septa of cells grown to an OD_600_ of ca. 0.3 (Figure 3C). Staining of the *msp1* mutant cells with anti-Msp2 also showed spots of fluorescence on opposite sites of the cell wall of the long clusters of cells grown to an OD_600_ of ca. 0.3 (Figure 3D). Staining with pre-immune serum as a negative control revealed no fluorescence in wild-type or *msp1* mutant cells (data not shown).

### Msp1 shows hydrolytic activity in zymogram assays

**Figure 4:**
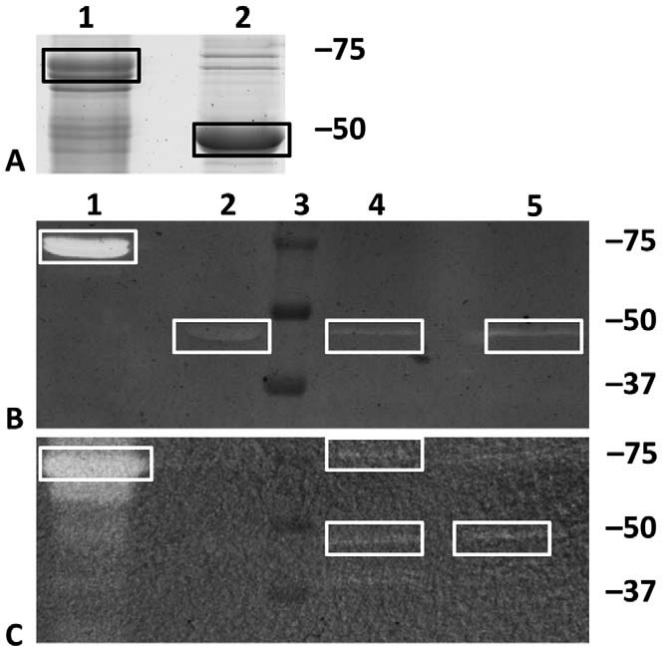
Msp1 and Msp2 on SDS-PAGE (A) and zymogram (B&C). Proteins were separated in 12% polyacrylamide SDS-PAGE gels (A,B&C), which were run in parallel with the same samples. 20 µg of recombinant Msp1 (lane 1) and Msp2 (lane 2) were put on the gels. Lane 3: molecular weight ladder. Lane 4 and 5: supernatant from wild-type LGG and *msp1* mutant, respectively. TCA treated wild-type LGG (B) and *msp1* mutant (C) were added as substrate. The gels for zymogram analysis were incubated overnight at 37°C in phosphate buffer containing 1 mM DTT. The boxes indicate the location of Msp1 and Msp2 protein.

To determine whether Msp1 and Msp2 have cell wall hydrolytic activity, both proteins were expressed in *E. coli* for purification. With the use of a histidine tag, the proteins were purified and analyzed on SDS-PAGE (Figure 4A). TCA-treated wild-type and *msp1* mutant cells were used as substrate in renaturing SDS-PAGE (zymogram). Msp1 revealed hydrolytic activity on wild-type (Figure 4B) and *msp1* mutant cells (Figure 4C), although the activity of the Msp1 protein was more pronounced on *msp1* mutant cell substrate (Figure 4BC). TCA treatment was necessary to remove exopolysaccharides and teichoic acids, to make PG more exposed. As Msp1 and Msp2 are secreted in high amounts by LGG wild-type [1] and as the Msp1 protein appears to be glycosylated [7], native proteins were also concentrated from spent culture supernatant. The concentrated supernatant proteins also seem to suggest hydrolytic activity of Msp1 and a protein of ca. 40 kDa (Figure 4BC). Supernatant of the *msp1* mutant expressing a C-terminally truncated Msp1 protein only showed activity around ca. 40 kDa, confirming that the C-terminal NlpC/P60 domain of Msp1 is crucial for its lytic activity observed around 70 kDa. The hydrolytic activity of the protein of ca. 40 kDa is possibly linked to the Msp2 protein, but so far no direct proof could be obtained. Recombinantly expressed Msp2 did not show a significant hydrolytic activity on zymograms (Fig. 4BC).

### Hydrolytic specificity of the Msp1 protein

**Figure 5:**
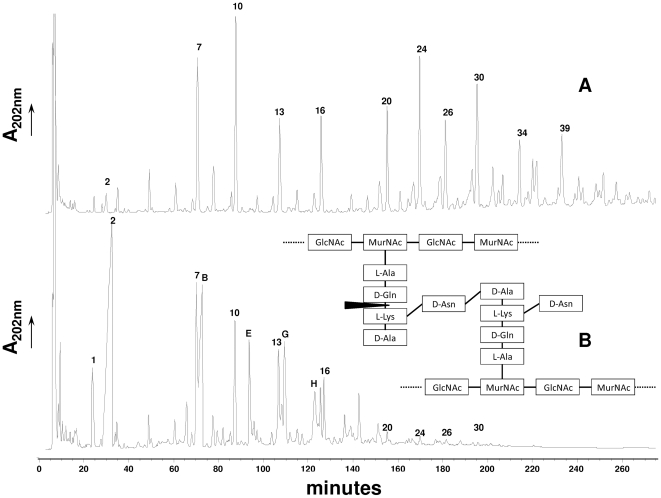
RP-HPLC separation profile of muropeptides from LGG digested with mutanolysin (A) and digested with mutanolysin and Msp1 (B). Peak numbers refer to Table 1. DS-di, disaccharide dipeptide (GlcNAc-MurNAc-L-Ala-D-Gln). The schematic structure of LGG peptidoglycan and site of cleavage of Msp1 is also represented. GlcNAc, N-acetylglucosamine; MurNAc, N-acetylmuramic acid.

To investigate the exact hydrolytic specificity of the Msp1 protein, we assayed the Msp1 activity on LGG PG. PG isolated from exponentially grown cells (OD_600_ 0.3) was treated with either mutanolysin alone or with mutanolysin and the Msp1 protein. The obtained muropeptide fragments were then separated by RP-HPLC (Figure 5). In total, 48 peaks were detected after RP-HPLC separation of the reduced muropeptides obtained from mutanolysin digestion of LGG PG (Figure 5A). Their structure was deduced from their mass determined by MALDI-TOF MS (Figure S1 and Table S2). When the PG was digested with both mutanolysin and Msp1 (Figure 5B), 42 peaks, including 19 peaks with new masses not present in the mutanolysin digest, were detected and identified by MS (Figure S1 and Table S2).

In the PG digested with mutanolysin and Msp1 (Figure 5B), a strong accumulation of disaccharide dipeptide (Peak 2, Table 1) and acetylated form of disaccharide dipeptide (peak B, Table 1) were observed. Also, a striking decrease of dimers (peaks 20, 24, 26, 30) and complete disappearance of trimers and tetramers (see Figure S1 and Table S2) is visible on Figure 5B. Concomitantly, new peaks (Peaks B, E, G, H) containing muropeptide fragments resulting from cleavage of disaccharide dipeptide were detected (Table 1). For example, the digestion of peak 20, a muropeptide dimer Tri-N-Tetra-N, by Msp1 results in peak 2 (DS-Di) and peak E (DS-Tri-N-A-K-N).

**Table 1:** Main muropeptides from LGG PG digested with or without Msp1.

Peak^a^	Proposed structure^b^	Observedm/z	Calculated^c^[M+Na]^+^	% of all peaks^d^^,^ e	
				Without Msp1	With Msp1
2	Di	720.28	720.29	2.26	15.19
7	Tri-N	962.33	962.43	6.21	6.62
10	Tetra-N	1033.39	1033.47	7.89	4.48
13	Tri-N(Ac)	1004.67	1004.44	3.94	4.43
16	Tetra-N(Ac)	1075.52	1075.48	4.12	2.78
20	Tri-N-Tetra-N	1954.70	1954.90	4.39	0.43
24	Tetra-N-Tetra-N	2025.89	2025.93	6.95	0.41
26	Tri-N-Tetra-N(Ac)	1996.83	1996.90	4.52	0.28
30	Tetra-N-Tetra-N(Ac)	2067.75	2067.94	6.67	0.15
34	Tetra-N-Tetra-N-Tetra-N	3018.15	3018.40	3.12	ND
39	Tetra-N-Tetra-N-Tetra-N(Ac)	3060.32	3060.41	4.28	ND
B	Di(Ac)	762.24	762.30	ND	1.91
E	Tri-N-(A-K)-N	1275.46	1275.60	ND	4.64
G	Tetra-N-(A-K)-N	1346.56	1346.65	ND	4.86
H	Tetra-N-(A-K)-N-(A-K)-N	1659.82	1659.82	ND	4.24

a Peak numbers refer to Figure 5 and Figure S1.

b Di, disaccharide dipeptide (L-Ala-D-iGln); Tri, disaccharide tripeptide (L-Ala-D-iGln-L-Lys); Tetra, disaccharide tetrapeptide (L-Ala-D-iGln-L-Lys-D-Ala); Disaccharide, GlcNAc-MurNAc; Ac, acetylation, iGln, isoglutamine; N, D-Asn; A, D-Ala; K, L-Lys.

c Sodiated molecular ions were the most abundant ones on MALDI-TOF mass spectra for all muropeptides.

d Percentage of each peak was calculated as the ratio of the peak area over the sum of areas of all the peaks identified in the corresponding chromatogram (see Figure 5).

e ND, non detected.

According to results obtained on the different monomers listed in Table 1, disaccharide with tetrapeptide stem peptide appeared as better substrates than disaccharide with tripeptide chain. Also, dimers and other multimers were better hydrolyzed than monomers (Table 1). Together, these data demonstrate the D-glutamyl-L-lysyl endopeptidase activity of the Msp1 protein (Figure 5).

## Discussion

In this work, we aimed to elucidate the physiological role of the major secreted proteins Msp1 and Msp2 (designated formerly as p75 and p40, respectively [1]) in the probiotic strain LGG. Although each of these proteins has been reported previously to show homology with cell wall hydrolases [1], the associated enzymatic activity and site and timing of action remained to be demonstrated. Interestingly, it was revealed recently that two similar proteins (LCABL_00230 and LCABL_02770) in the related *L. casei* BL23 strain possess hydrolase activity against cell wall components [8]. However, there are apparent differences in the primary structure between these proteins and the LGG Msp1 and Msp2 proteins. For instance, a different repetitive sequence (residues 272 to 332) can be found in *L. casei* versus *L. rhamnosus*. The Msp1-like protein of *L. casei* contains an Ala-Ala-Ala-Ser repeat and a Pro-rich region, whereas Msp1 from *L. rhamnosus* contains an Ala-Ala-Ala-Ser-Gln sequence [8]. The function of these repetitive sequences is currently unknown, but repetitive sequences are often involved in cell wall binding of cell wall hydrolases [6]. In addition, the proteins differ remarkably around residue 100. Recently, we could show that the Msp1 protein of LGG is glycosylated at the serine residues in T_101_V E T P S S A_108_. In *L. casei*, these serine residues are absent in the same region (T_101_V Q A P E Q Y_108_) [7]. Moreover, differences in the total complement of PG hydrolases, some of which could have similar biological, redundant or complementary functions are expected based on the genome sequences [9], [10].

To provide evidence that the Msp1 protein of LGG can indeed behave as cell wall hydrolase, we used a triple approach, i.e. (1) the characterization of a knock-out mutant to study its functional importance in live bacterial cells, (2) immunofluorescence microscopy to study the preferred site (and timing) of action, and (3) the use of purified native or recombinant protein to study the hydrolytic capacity and enzymatic activity. As our results showed, inactivation of the *msp1* gene in LGG resulted in an intriguing phenotype, as the daughter cell separation was drastically hampered. This mutant lacking the NlpC/P60 domain of Msp1 produced long chains of unseparated cells, as visualized with phase-contrast and TEM microscopy. Moreover, immunofluorescence revealed that Msp1 accumulates specifically at the septum of exponentially growing cells. Previously, the related NlpC/P60 enzymes were demonstrated to be involved in cell separation in some *Firmicutes*, i.e. *Listeria monocytogenes*, *Staphylococcus aureus* and multiple *Bacillus* species [5].

By means of zymogram analysis, the common technique for assessing cell wall hydrolase activity [11], [12], [13], we were able to confirm this enzymatic property for Msp1. Using purified PG, the hydrolytic specificity of the Msp1 protein was subsequently determined as being a D-glutamyl-L-lysyl endopeptidase, similar to the NlpC/P60 protein YjgB in *Lactococcus lactis* [11] and Lc2770 in *L. casei* BL23 (Regulski *et al.*, submitted back to back with this manuscript).

Despite our repeated attempts, we failed to construct an *msp2* mutant using an established protocol for LGG [9], [14], [15], suggesting that Msp2 may be essential for the growth and cell division of LGG. This is remarkable, as an *msp2* mutant could be constructed in *L. casei* BL23 [8]. This suggests that the complement of PG hydrolases in *L. casei* BL23 contains enzymes with (partially) redundant activity of Msp2, which are absent in LGG. On the other hand, our immunofluorescence data suggest a possible role for Msp2 in early stage cell septum formation. This is supported by the observation that Msp2 is localized at two positions on opposite sites of the cell. In addition, experiments with recombinantly expressed Msp2 or purified native Msp2 from LGG supernatant samples did not indicate a large hydrolytic PG degrading activity enzymatic activity Future experiments are aimed at characterizing the exact hydrolytic and enzymatic activity of Msp2 and its possible role in priming septum initiation.

The data presented in this manuscript indicate an important physiological role for the Msp1 protein of LGG, which appears to be a D-glutamyl-L-lysyl endopeptidase essential for a normal separation of LGG cells. The function of the Msp2 remains to be clarified, but the immunofluorescent data indicate a role in early septum formation. Intriguingly, both proteins are documented important probiotic effectors [1], [3], suggesting that these proteins perform a dual role.: i.e. their primary function as PG hydrolase (these data) and a secondary function as probiotic effectors regulating intestinal epithelial cell homeostasis [1], [2], [3]. Knowledge on the bacterial physiological role of these proteins also helps our understanding of why and when LGG secretes these probiotic proteins and will contribute to define the conditions for the optimal performance of these proteins as probiotic effectors. Moreover, these two functions may well be interconnected through the enzymatic action of these two proteins with glycocalyx/mucosal components of a similar composition to that of PG. Further experiments should elucidate the exact signaling capacity of these proteins in interaction with intestinal epithelial cells.

## Materials and Methods

### Bacterial strains, media and growth conditions

LGG and its derivatives were routinely grown at 37°C in de Man-Rogosa-Sharpe (MRS) medium (Difco) under static conditions. Lactobacilli AOAC medium (Difco) was also used in this study. For the growth of the *msp1* mutant and the complementation construct, 5 µg/ml of erythromycin (Ery) and 5 µg/ml of chloramphenicol (Cm) were applied respectively. *E. coli* strains were grown in Luria-Bertani (LB) medium at 37°C with shaking. When required erythromycin (100 µg/ml), ampicillin (100 µg/ml), or kanamycin (50 µg/ml) was added to the growth media. *E. coli* strains TOP10F (Invitrogen) and BL21 (DE3)/pLysS served as hosts for DNA cloning and protein expression, respectively.

### DNA manipulations

Routine molecular biology techniques were performed according to standard procedures [16]. Restriction and modifying enzymes (from New England Biolabs and Roche) were used as recommended by the manufacturers. Plasmid DNA was prepared from *E. coli* by using QIAGEN Miniprep kits. Chromosomal DNA was isolated from LGG as described previously [14].

### Construction of the msp1 mutant

When the construction of the mutant was initiated, the genome sequence of LGG was not yet available, although the amino acid sequence of Msp1 (p75) was determined by Yan *et al.* [1]. A protein BLAST resulted in the identification of the Msp1 homologue of *L. casei* ATTC 334 (YP_805583.1). By designing primers based on the sequence of *L. casei*, the gene sequence of *msp1* was determined. Primers (Pro-0997 and Pro-0998) were used to amplify an internal fragment of the *msp1* gene of approximately 1000 bp (for primer sequences see Table S1). The resulting internal fragment was cloned into pCRII-TOPO vector (Invitrogen). The resulting vector was further digested with *Eco*RI and ligated in vector pFAJ5301, an erythromycin-resistant derivative of pUC18 [15]. The ligation product was used to transform *E. coli* TOP10F. This suicide vector was then electroporated to LGG as described previously [14]. Integration of the plasmid by single homologues recombination was confirmed with PCR and SDS-PAGE and designated CMPG10200.

### Complementation of the knock-out msp1 mutant

Using primers Pro-1440 and Pro-1441, a functional *msp1* gene was amplified by PCR. The resulting fragment was introduced in the high copy pLAB1301 [17]-derived vector CMPG10212, in which the Ery resistance gene was removed by a restriction digest with *Sna*BI and *Sma*I and replaced by the ‘boosted’ Cm resistance marker, encoded by an *Dra*I-*Sma*I cut DNA fragment from plasmid pGIZ850 [18]. The resulting vector was subcloned in *E. coli* TOP10F and further electroporated to the LGG *msp1* mutant and to the wild-type as a control as described previously [14]. The strain was designated CMPG10203.

### Plasmids for msp2 mutant construction

Primers Pro-0985 and Pro-0986 were used to amplify an internal sequence of the *msp2* gene of approximately 1000 bp. The resulting internal fragment was cloned into pCRII-TOPO vector (Invitrogen). The resulting vector was further digested with *Eco*RI and ligated in vector pFAJ5301 [15]. The ligation product was used to transform *E. coli* TOP10F. This suicide vector was then electroporated to LGG [14]. Four independant electroporations were performed. Although the transformation efficiency was similar (i.e. 10^4^ CFU/µg DNA) as observed previously [14], and which corresponds to an average recombinantion frequency of 3×10^2^ per µg DNA, no insertional mutants could be obtained.

### Msp1 and Msp2 from LGG spent culture supernatant

For SDS-PAGE and Western blot, bacteria were grown for 24 h in AOAC-medium. After centrifugation (6000 g, 20 min), proteins were precipitated from supernatant by incubation at 4°C for 30 min in the presence of trichloroacetic acid (TCA) (20% final concentration). After centrifugation (150000 g, 20 min), precipitated proteins were washed twice with cold acetone. Pellet was air-dried and proteins were resuspended in lysis buffer (2 M thio-urea, 7 M urea, 4% 3[(3-Cholamidopropyl)dimethylammonio]-propanesulfonic acid (CHAPS), 2% dithiothreitol (DTT)). Supernatant samples were subsequently run by SDS-PAGE. Msp1 and Msp2 were identified by Edman degradation as described previously [19] or by Western blot analysis with specific antisera.

To investigate the enzymatic activity, proteins were purified in their native form from spent culture supernatant by using Vivaspin centrifugal concentrators (Sartorius Stedim biotech GmbH, 37070 Goettingen Germany) (Molecular Weight Cutt Off 50 000 Da) followed by dialysis against phosphate buffered saline (PBS).

### Partial purification of the Msp1 protein

The Msp1 protein was partially purified by cationic exchange chromatography as described previously [1]. Partial purification of the Msp1 protein was performed by loading culture supernatant onto SP Sepharose HighPerfomance (GE Healthcare), equilibrated with 60 mM lactate buffer pH 4.0. Bound proteins were eluted using lactate buffer containing sequential NaCl concentrations (100–1000 mM). Fractions positive for the presence of Msp1 were identified using SDS-PAGE and spin concentrated using Vivaspin filters with MW cut off 10.000 Da.

### Cloning, expression, and purification of recombinant Msp1 and Msp2

The coding sequence for *msp1* (*LGG_00324*) or *msp2* (*LGG_00031*), without the N-terminal signal peptide sequence, was PCR amplified from LGG genomic DNA using pairs of flanking 5′- and 3′-end oligoprimers, one containing an *Eco*RI restriction site (5′- TACCGTTTCGAATTCGACAGGGACGGTC for *msp1* and 5′-ACCGGTTTTGAATTCCACAAGTGCCAGC for *msp2*; *Eco*RI site is underlined) and another containing an *Xho*I restriction site (5′- GATCATTAAGCCTCGAGTGACGGGCGAAC for *msp1* and 5′- TAAGGGTGGGTAACTCGAGCCGGTGGATG for *msp2*; *Xho*I site is underlined) (Oligomer, Finland). PCR fragments were cleaved with *Eco*RI and *Xho*I restriction endonucleases and ligated into the pET28b+ expression vector (Novagen). The resulting recombinant plasmids (pKTH5316 for *msp1* and pKTH5317 for *msp2*) were propagated in the *E. coli* strain BL21 (DE3)/pLysS for the intracellular expression of 48.4-kDa (Msp1) and 41.5-kDa (Msp2) C-terminal hexahistidine-tagged proteins. Recombinant Msp1 and Msp2 also consist of an additional seven amino acids at the N-terminus and two amino acids before the C-terminal hexahistidine-tag that derive from the coding sequence of the expression vector. Protein production was performed as described previously [9]. In brief, *E. coli* cells having either the pKTH5316 or pKTH5317 plasmids were grown at 37°C in LB-kanamycin (50 µg/ml) broth. After reaching the mid-log phase, soluble protein production was induced by 1 mM isopropyl β-D-1-thiogalactopyranoside (IPTG) for 3 h at 37°C for Msp1 or at room temperature for Msp2. Cells were then recovered by centrifugation and broken by sonication in lysis buffer (50 mM NaH_2_PO_4_ [pH 8.0], 300 mM NaCl, 10 mM imidazole). Cell suspensions were clarified by centrifugation and membrane-filtering (0.45-µm pore-size) and applied to a nickel nitrilotriacetic acid (Ni-NTA) agarose (Qiagen) column from which, after rinsing with wash buffer (50 mM NaH_2_PO4 [pH 8.0], 300 mM NaCl, 20 mM imidazole), the Msp1 and Msp2 proteins were then eluted with elution buffer (50 mM NaH_2_PO_4_ [pH 8.0], 300 mM NaCl, 250 mM imidazole) as described previously [9]. Purified Msp1 or Msp2 was buffer exchanged to 10 mM Tris-HCl (pH 8.0) with a Bio-Rad EconoPac 10 DG desalting column and then concentrated using a 30-kDa Microsep filter (Pall Life Sciences). SDS-PAGE was used to visualize protein purity and the absorbance at 280 nm was measured to estimate the protein concentration.

### Generation of Msp1 and Msp2-specific polyclonal antisera

Polyclonal antiserum specific for Msp1 or Msp2 was raised in rabbits as described previously [9]. Briefly, a 1 ml volume of 400 µg purified recombinant protein (Msp1 or Msp2) in Freund's complete adjuvant (1∶1 mixture) was first injected subcutaneously, which was then followed by 1 ml subcutaneous booster injections of 200 µg Msp1 or Msp2 protein in Freund's incomplete adjuvant (1∶1 mixture) at 3-week intervals during a 9-week period. Blood samples were recovered 14 days after the final booster injection, and the antiserum was prepared according to an established procedure [20].

### Western Blot Analysis

The proteins present in each crude or purified preparation of LGG wild-type spent culture supernatant were separated by SDS-PAGE in NuPage 10% BIS-tris gels. TCA precipitated excreted protein samples were loaded onto the wells of the SDS-containing gel after adding buffer and reducing agent according to the manufacturer's instructions. Equivalent amounts, determined by a bicinchoninic acid (BCA) assay, of protein were separated from the LGG wild-type strain and the *msp1* mutant strain (CMPG10200). The proteins were electroblotted onto polyvinyldifluoride (PVDF) membrane. The membranes were blocked with 3% bovine serum albumine (BSA) in TBS (20 mM Tris–HCl, 500 mM NaCl, pH 7.5) and incubated at room temperature with Msp1 antiserum (1∶2000) for 1 h. After several washings with TBS containing 0.05% Tween-20, blots were incubated for 1 h with goat anti-rabbit antibodies conjugated with alkaline phosphatase (Roche) at a dilution of 1∶1000. Detection was performed with nitro blue tetrazolium and bromo-chloro indolyl phosphate as substrate and the blue coloring reaction was monitored.

### Electron microscopy

LGG wild-type and CMPG10200 mutant cells were grown to stationary phase in AOAC medium, washed once with PBS and then diluted (OD600 = 1.0) in the same buffer. Formvar-carbon-coated copper grids were floated for 30 minutes on droplets of the LGG and CMPG10200 cells. Next, the grids were washed with distilled water, and negatively stained with a mixture of 1.8% methylcellulose–0.4% uranyl acetate. To obtain images, the grids were examined with JEOL 1200 EX II transmission electron microscope.

### Immunofluorescence microscopy

The cellular localization of Msp1 and Msp2 was determined by immunofluorescence assays. Hereto, LGG wild-type and mutant strains were grown until exponential phase (optical density (OD_600_) of ±0.3) in AOAC broth. Cells were fixed for 1 h at room temperature in 2.5% formaldehyde and 0.05% gluteraldehyde. The cells were washed once with PBS and incubated for 10 min in PBS containing 0.5% blocking reagent (Boehringer). The samples were then incubated in blocking buffer containing anti-Msp1 and anti-Msp2 rabbit polyclonal antiserum or pre-immune serum (1∶400 dilution) for 30 min at room temperature, washed three times with PBS, and subsequently incubated in blocking buffer containing goat anti-rabbit IgG (1∶200 dilution in PBS) conjugated to Alexa Fluor 488 (Invitrogen) for 30 min at room temperature. The cells were washed three times with PBS to remove unbound antibodies and the cells were transferred to a poly-L-lysine-coated microscopic slide and a coverslip was mounted. Samples were visualized with a Zeiss Axio Imager Z1, equipped with an AxioCam MRm Rev.3 monochrome digital camera. The samples were imaged with a ‘Plan-Neofluar’ 100×/1.3 Oil Ph3 objective. Images were analyzed with the supplied AxioVision Rel.4.6 software making overlays of phase-contrast and fluorescent images.

### SDS-PAGE and zymogram analysis

The cell wall hydrolyzing activity was investigated by zymogram analysis as described previously by Lepeuple *et al.* [21]. SDS-PAGE was performed with 10% (w/v) polyacrylamide separating gels (NuPage, Invitrogen). Autoclaved LGG cells treated with 10% TCA were added to the gels as enzyme substrates at 0.4% (w/v) and the gels were loaded with 10 to 20 µg of protein samples. After sample migration, the gels were washed for 30 min in deionized H_2_O at room temperature and then incubated in a 50 mM phosphate buffer, pH 6.2, 1 mM dithiothreitol (DTT) containing 0.1% (v/v) Triton X-100, overnight at 37°C. The gels were subsequently washed for 30 min in deionized H_2_O, then stained with 0.1% Methylene Blue in 0.01% (w/v) KOH for 2 h at room temperature and destained in deionized H_2_O.

### Peptidoglycan extraction and structural analysis

PG from LGG was prepared as described previously [12] with some modifications. Additional nucleic acids were removed by treatment with RNase (100 µg/ml) and DNase (100 µg/ml). The pellet containing PG was treated with 10% TCA for 24 h at 4°C to eliminate exopolysaccharides and teichoic acids, washed twice with 0.25 M Tris/HCl, pH 8.0, and then four times with deionized H_2_O. Subsequently, the material was treated with 48% hydrofluoric overnight at 4°C to further eliminate exopolysaccharides and teichoic acids and washed as described above. The final pellet was freeze-dried and stored at −20°C until further analysis.

Purified PG (2 mg dry weight) was first digested with mutanolysin from *Streptomyces globisporus* (Sigma) (2500 U/ml) for 19 h in 25 mM sodium phosphate buffer at 37°C. The resulting soluble muropeptides were reduced with sodium borohydride. The mixture was adjusted to pH 6.5 and supplemented with 5 µM DTT. To determine the hydrolytic specificity of Msp1, 80 µg of pure recombinant His_6_-tagged Msp1 was incubated with 200 µl of the muropeptide mixture at 37°C during 24 h. Control sample was incubated in the same conditions without Msp1. The samples were boiled for 3 min. The products were separated by reverse phase-high-pressure liquid chromatography (RP-HPLC) as described previously [22]. Muropeptides were analyzed without desalting by MALDI-TOF mass spectrometry (MS) using a Voyager-DE STR mass spectrometer (Applied Biosystems) as reported previously [22].

## Supporting Information

Figure S1 **RP-HPLC separation profile of muropeptides obtained from LGG PG digested by mutanolysin (A) and by mutanolysin and recombinant Msp1 (B).**
(TIF)

Table S1 **Primers sequences used in this study.**
(DOC)

Table S2 **Structures, molecular masses and proportions of muropeptides obtained from LGG PG digested by mutanolysin and by mutanolysin and recombinant Msp1 protein.**
(DOC)

## References

[1] Yan F, Cao H, Cover TL, Whitehead R, Washington MK (2007). Soluble proteins produced by probiotic bacteria regulate intestinal epithelial cell survival and growth.. Gastroenterology.

[2] Yan F, Polk DB (2002). Probiotic bacterium prevents cytokine-induced apoptosis in intestinal epithelial cells.. J Biol Chem.

[3] Yan F, Cao H, Cover TL, Washington MK, Shi Y (2011). Colon-specific delivery of a probiotic-derived soluble protein ameliorates intestinal inflammation in mice through an EGFR-dependent mechanism.. J Clin Invest.

[4] Weidel W, Pelzer H (1964). Bagshaped macromolecules–a new outlook on bacterial cell walls.. Adv Enzymol Relat Areas Mol Biol.

[5] Layec S, Decaris B, Leblond-Bourget N (2008). Diversity of Firmicutes peptidoglycan hydrolases and specificities of those involved in daughter cell separation.. Res Microbiol.

[6] Vollmer W, Joris B, Charlier P, Foster S (2008). Bacterial peptidoglycan (murein) hydrolases.. FEMS Microbiol Rev.

[7] Lebeer S, Claes IJ, Balog CI, Schoofs G, Verhoeven TL (2011). The major secreted protein Msp1/p75 is 1 O-glycosylated in *Lactobacillus rhamnosus* GG.. Microbial Cell Factories.

[8] Bäuerl C, Perez-Martinez G, Yan F, Polk DB, Monedero V (2010). Functional analysis of the p40 and p75 proteins from *Lactobacillus casei* BL23.. J Mol Microbiol Biotechnol.

[9] Kankainen M, Paulin L, Tynkkynen S, von Ossowski I, Reunanen J (2009). Comparative genomic analysis of *Lactobacillus rhamnosus* GG reveals pili containing a human-mucus binding protein.. Proc Natl Acad Sci U S A.

[10] Maze A, Boel G, Zuniga M, Bourand A, Loux V (2010). Complete genome sequence of the probiotic *Lactobacillus casei* strain BL23.. J Bacteriol.

[11] Redko Y, Courtin P, Mezange C, Huard C, Chapot-Chartier MP (2007). *Lactococcus lactis* gene *yjgB* encodes a gamma-D-glutaminyl-L-lysyl-endopeptidase which hydrolyzes peptidoglycan.. Appl Environ Microbiol.

[12] Meyrand M, Boughammoura A, Courtin P, Mezange C, Guillot A (2007). Peptidoglycan N-acetylglucosamine deacetylation decreases autolysis in *Lactococcus lactis*.. Microbiology.

[13] Prado Acosta M, Mercedes Palomino M, Allievi MC, Sanchez Rivas C, Ruzal SM (2008). Murein hydrolase activity in the surface layer of *Lactobacillus acidophilus* ATCC 4356.. Appl Environ Microbiol.

[14] De Keersmaecker SC, Braeken K, Verhoeven TL, Perea Velez M, Lebeer S (2006). Flow cytometric testing of green fluorescent protein-tagged *Lactobacillus rhamnosus* GG for response to defensins.. Appl Environ Microbiol.

[15] Lebeer S, De Keersmaecker SC, Verhoeven TL, Fadda AA, Marchal K (2007). Functional analysis of *luxS* in the probiotic strain *Lactobacillus rhamnosus* GG reveals a central metabolic role important for growth and biofilm formation.. J Bacteriol.

[16] Sambrook J, Fritsch EF, Maniatis T (1989). Molecular cloning. A Laboratory Manual.

[17] Josson K, Scheirlinck T, Michiels F, Platteeuw C, Stanssens P (1989). Characterization of a gram-positive broad-host-range plasmid isolated from *Lactobacillus hilgardii*.. Plasmid.

[18] Goffin E, Herbiet L, Pouthier D, Pochet JM, Lafontaine JJ (2004). Vancomycin and ciprofloxacin: systemic antibiotic administration for peritoneal dialysis-associated peritonitis.. Perit Dial Int.

[19] Van den Steen PE, Proost P, Grillet B, Brand DD, Kang AH (2002). Cleavage of denatured natural collagen type II by neutrophil gelatinase B reveals enzyme specificity, post-translational modifications in the substrate, and the formation of remnant epitopes in rheumatoid arthritis.. Faseb J.

[20] Harlow E, Lane D (1988). Antibodies: A Laboratory Manual.

[21] Lepeuple AS, Van Gemert E, Chapot-Chartier MP (1998). Analysis of the bacteriolytic enzymes of the autolytic *Lactococcus lactis* subsp. *cremoris* strain AM2 by renaturing polyacrylamide gel electrophoresis: identification of a prophage-encoded enzyme.. Appl Environ Microbiol.

[22] Courtin P, Miranda G, Guillot A, Wessner F, Mezange C (2006). Peptidoglycan structure analysis of *Lactococcus lactis* reveals the presence of an L,D-carboxypeptidase involved in peptidoglycan maturation.. J Bacteriol.

