# Supplementary figures and images for: Genetic and Biochemical Characterization of the Cell Wall Hydrolase Activity of the Major Secreted Protein of Lactobacillus rhamnosus GG
Source: PLoS One. 2012 Feb 16;7(2):e31588. doi: 10.1371/journal.pone.0031588 (PMC3281093; doi:10.1371/journal.pone.0031588)

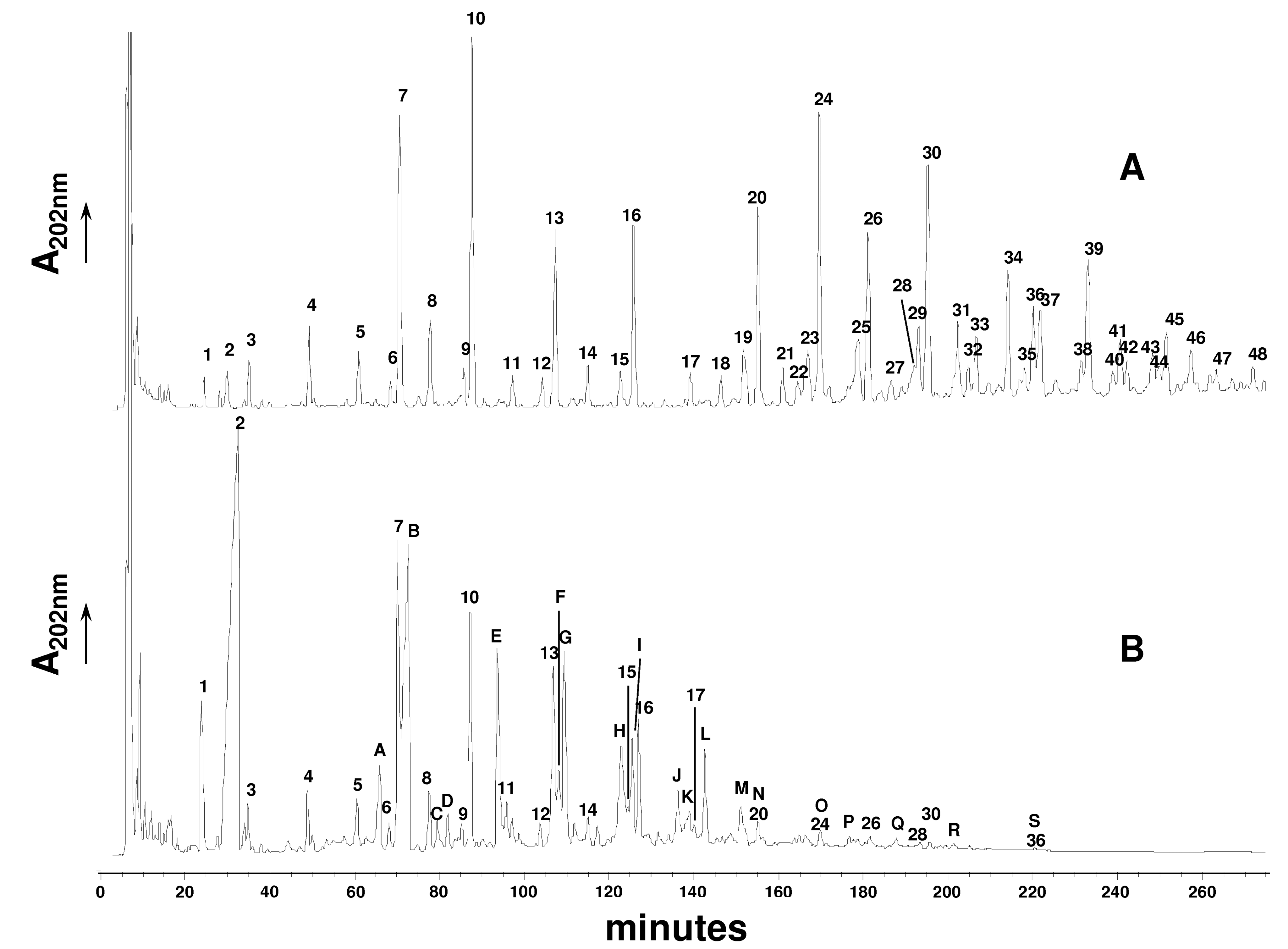

Supplement: Figure S1 — RP-HPLC separation profile of muropeptides obtained from LGG PG digested by mutanolysin (A) and by mutanolysin and recombinant Msp1 (B). (TIF) [file pone.0031588.s001.tif]
