# Supplementary material for: Genetic and Biochemical Characterization of the Cell Wall Hydrolase Activity of the Major Secreted Protein of Lactobacillus rhamnosus GG
Source: PLoS One. 2012 Feb 16;7(2):e31588. doi: 10.1371/journal.pone.0031588 (PMC3281093; doi:10.1371/journal.pone.0031588)
Supplement: Table S1 — Primers sequences used in this study. (DOC) [file pone.0031588.s002.doc]

Table S1: Primers sequences used in this study

| Primer | Sequence (5’-3’) |
| --- | --- |
| Pro-0985 (EcoRI) | ATGAATTCTTGGTTGCAGCAGTTACCTTAGCG |
| Pro-0986 (EcoRI) | ATGAATTCGAAGACGATGATGGAACCTGCTGC |
| Pro- 0997 (EcoRI) | ATGAATTCACAGGGACGGTCAGTTACAAATCC |
| Pro- 0998 (EcoRI) | ATGAATTCGTTGTTCGGCAATGGCAATCACTG |
| Pro-1440 (BamHI) | ATCGATCGACGTATAAGTTGAAGGCATACCTG |
| Pro-1441 (BamHI) | ATCGATCGTACAAAACCAGACAGTTCATGCAG |
