# Supplementary material for: Genetic and Biochemical Characterization of the Cell Wall Hydrolase Activity of the Major Secreted Protein of Lactobacillus rhamnosus GG
Source: PLoS One. 2012 Feb 16;7(2):e31588. doi: 10.1371/journal.pone.0031588 (PMC3281093; doi:10.1371/journal.pone.0031588)
Supplement: Table S2 — Structures, molecular masses and proportions of muropeptides obtained from LGG PG digested by mutanolysin and by mutanolysin and recombinant Msp1 protein. (DOC) [file pone.0031588.s003.doc]

Table S1: Structures, molecular masses and proportions of muropeptides obtained from LGG PG digested by mutanolysin and by mutanolysin and recombinant Msp1

| Peak*^a^* | Proposed structure*^b^* | Observed  m/z | Calculated*^c^*  [M+Na]^+^ | % of all peaks*^d,e^* | |
| --- | --- | --- | --- | --- | --- |
|  |  |  |  | Without Msp1 | With Msp1 |
| 1 | Tri missing GlcNac | 645.12 | 645.31 | 0.45 | 3.02 |
| 2 | Di | 720.31 | 720.29 | 0.86 | 27.36 |
| 3 | Tri | 848.45 | 848.39 | 0.75 | 0.72 |
| 4 | Tetra | 919.33 | 919.42 | 1.32 | 0.98 |
| 5 | Tri-D | 963.33 | 963.41 | 1.05 | 0.94 |
| 6 | Tri (Ac) | 890.31 | 890.40 | 0.42 | 0.51 |
| 7 | Tri-N | 962.33 | 962.43 | 6.21 | 6.62 |
| 8 | Tetra-D | 1034.36 | 1034.43 | 1.95 | 1.18 |
| 9 | Tetra (Ac) | 961.27 | 961.43 | 0.75 | 0.50 |
| 10 | Tetra-N | 1033.39 | 1033.47 | 7.89 | 4.48 |
| 11 | Tri-D (Ac) | 1005.45 | 1005.42 | 0.76 | 1.57 |
| 12 | Tri-N (Ac) | 1004.57 | 1004.44 | 0.71 | 0.47 |
| 13 | Tri-N(Ac) | 1004.67 | 1004.44 | 3.94 | 4.43 |
| 14 | Tetra-D (Ac) | 1076.46 | 1076.46 | 0.97 | 0.77 |
| 15 | Tetra-N (Ac) | 1075.48 | 1075.48 | 0.89 | 0.68 |
| 16 | Tetra-N(Ac) | 1075.52 | 1075.48 | 4.12 | 2.78 |
| 17 | Tri-N-Tetra | 1840.84 | 1840.85 | 0.72 | 0.55 |
| 18 | Tri-D-Tetra-N | 1883.93 | 1883. | 0.74 | ND |
| 19 | Tetra-N-Tetra-N | 1911.86 | 1911.89 | 2.05 | ND |
| 20 | Tri-N-Tetra-N | 1954.70 | 1954.90 | 4.39 | 0.53 |
| 21 | Tetra-D-Tetra (Ac) | 1954.91 | 1954.88 | 0.91 | ND |
| 22 | Tetra-N-Tetra-N missing GlcNac | 1822.74 | 1822.85 | 0.73 | ND |
| 23 | Tri-N-Tetra (Ac) | 1882.88 | 1882.86 | 2.18 | ND |
| 24 | Tetra-N-Tetra-N | 2025.89 | 2025.93 | 6.95 | 0.41 |
| 25 | Tetra-N-Tetra (Ac) | 1953.84 | 1953.90 | 2.55 | ND |
| 26 | Tri-N-Tetra-N(Ac) | 1996.83 | 1996.90 | 4.52 | 0.28 |
| 27 | Tri-N-Tetra-N (Ac) | 1996.80 | 1996.90 | 0.73 | ND |
| 28 | Tetra-N-Tetra-N (Ac) missing GlcNac | 1864.60 | 1864.86 | 1.54 | 0.13 |
| 29 | Tetra-N-Tetra-N (Ac) | 2067.75 | 2067.94 | 2.09 | ND |
| 30 | Tetra-N-Tetra-N(Ac) | 2067.75 | 2067.94 | 6.67 | 0.15 |
| 31 | Tri-N-Tetra-N-Tetra-N | 2946.99 | 2947.36 | 2.58 | ND |
| 32 | Tri-N-Tetra-N (2-Ac) | 2039.01 | 2038.91 | 0.89 | ND |
| 33 | Tri-N-Tetra-N (2-Ac) | 2038.90 | 2038.91 | 1.60 | ND |
| 34 | Tetra-N-Tetra-N-Tetra-N | 3018.15 | 3018.40 | 3.12 | ND |
| 35 | Tetra-N-Tetra-N (2-Ac) | 2109.99 | 2109.95 | 0.80 | ND |
| 36 | Tetra-N-Tetra-N (2-Ac) | 2109.88 | 2109.95 | 2.45 | 0.05 |
| 37 | Tetra-N-Tetra-N-Tetra (Ac) | 2946.31 | 2946.37 | 2.83 | ND |
| 38 | Tetra-N-Tetra-N-Tetra-N (Ac) | 3060.38 | 3060.41 | 0.92 | ND |
| 39 | Tetra-N-Tetra-N-Tetra-N(Ac) | 3060.32 | 3060.41 | 4.28 | ND |
| 40 | Tri-N-Tetra-N-Tetra-N (2-Ac) | 3031.31 | 3031.38 | 0.80 | ND |
| 41 | Tri-N-Tetra-N-Tetra-N (2-Ac) | 3031.31 | 3031.38 | 1.77 | ND |
| 42 | Tetra-N-Tetra-N-Tetra-N-Tetra-N | 4010.44 | 4010.87 | 0.92 | ND |
| 43 | Tri-N-Tetra-N-Tetra-N-Tetra-N (Ac) | 3981.90 | 3981.84 | 1.43 | ND |
| 44 | Tetra-N-Tetra-N-Tetra-N (2-Ac) | 3102.50 | 3102.42 | 0.89 | ND |
| 45 | Tetra-N-Tetra-N-Tetra-N (2-Ac) | 3102.31 | 3102.42 | 1.89 | ND |
| 46 | Tetra-N-Tetra-N-Tetra-N-Tetra-N (Ac) | 4052.72 | 4052.88 | 1.43 | ND |
| 47 | Tri-N-Tetra-N-Tetra-N-Tetra-N (2-Ac) | 4023.65 | 4023.85 | 0.80 | ND |
| 48 | Tetra-N-Tetra-N-Tetra-N-Tetra-N (2-Ac) | 4094.65 | 4094.89 | 0.77 | ND |
| A | Di (Ac) | 762.24 | 762.30 | ND | 3.02 |
| B | Di(Ac) | 762.24 | 762.30 | ND | 1.91 |
| C | Tri-D-2 (Ac) | 1204.58 | 1204.56 | ND | 0.67 |
| D | Tri-N-2 | 1161.56 | 1161.56 | ND | 0.70 |
| E | Tri-N-(A-K)-N | 1275.46 | 1275.60 | ND | 4.64 |
| F | Tri-N-2-N-2-N | 1588.73 | 1588.78 | ND | 2.03 |
| G | Tetra-N-(A-K)-N | 1346.56 | 1346.65 | ND | 4.86 |
| H | Tetra-N-(A-K)-N-(A-K)-N | 1659.82 | 1659.82 | ND | 4.24 |

*^a^*Peak numbers refer to Fig. 5 and supplemental Fig. S2.

*^b^*Di, disaccharide dipeptide (L-Ala-D-iGln); Tri, disaccharide tripeptide (L-Ala-D-iGln-L-Lys); Tetra, disaccharide tetrapeptide (L-Ala-D-iGln-L-Lys-D-Ala); Disaccharide, GlcNAc-MurNAc; Ac, acetylation, iGln, isoglutamine; N, D-Asn; A, D-Ala; K, L-Lys.

*^c^*Sodiated molecular ions were the most abundant ones on Maldi-Tof mass spectra for all muropeptides.

*^d^*Percentage of each peak was calculated as the ratio of the peak area over the sum of areas of all the peaks identified in the corresponding chromatogram (see supplemental Fig. S2).

*^e^*ND, non detected.
